# Rheumatoid factor and falsely elevated results in commercial immunoassays: data from an early arthritis cohort

**DOI:** 10.1007/s00296-021-04865-9

**Published:** 2021-05-04

**Authors:** Johanna E. Gehin, Rolf A. Klaasen, Ellen S. Norli, David J. Warren, Silje W. Syversen, Guro L. Goll, Trine Bjøro, Tore K. Kvien, Maria D. Mjaavatten, Nils Bolstad

**Affiliations:** 1 Department of Medical Biochemistry, Oslo University Hospital-Radiumhospitalet, Box 4953 Nydalen, 0424 Oslo, Norway; 2 Faculty of Medicine, University of Oslo, Oslo, Norway; 3 Division of Rheumatology and Research, Diakonhjemmet Hospital, Oslo, Norway; 4 Department of Rheumatology, Martina Hansens Hospital, Sandvika, Norway

**Keywords:** Rheumatoid factor, Immunoassay, Interference, Rheumatoid arthritis, Heterophilic antibodies

## Abstract

The aim of the study was to  assess RF cross-reactivity to animal antibodies used in immunoassays, and to test if selected commercial immunoassays are vulnerable to interference from RF, causing false test results. Our study included samples from patients with RF-positive rheumatoid arthritis (RA) and controls (patients with RF-negative RA and psoriatic arthritis), included in an early arthritis-cohort. Reactivity to mouse IgG1, mouse IgG2a, rabbit IgG, bovine IgG, sheep/goat IgG and human IgG was analysed using in-house interference assays. RF-positive sera with strong reactivity to mouse IgG1 were analysed in three commercial immunoassays. To reveal interference, results before and after addition of blocking aggregated murine IgG1 were compared. Samples from 124 RF-positive RA patients and 66 controls were tested. We found considerably stronger reactivity toward animal antibodies, particularly mouse IgG1 (73% vs. 12%) and rabbit IgG (81% vs. 6%), in sera from RF-positive RA-patients compared to controls (*p* < 0.001). After selecting samples for testing in commercial assays, interference was revealed in 6/30 sera in the Architect β-hCG assay, 7/10 sera in the 27-plex cytokine assays, and in 2/33 samples in the Elecsys Soluble Transferrin Receptor assay. Our study revealed considerable RF reactivity to animal antibodies used in immunoassays and RF was associated with falsely elevated results in immunoassays used in clinical care and research. Clinicians, laboratorians, researchers and assay manufacturers must be alert to the risk of falsely elevated test results in RF-positive RA patients.

**Supplementary Information:**

The online version contains supplementary material available at 10.1007/s00296-021-04865-9.

## Introduction

Immunoassays are widely used to measure analytes in clinical practice for diagnostics and disease monitoring, as well as in research. The technology relies on animal antibodies, commonly mouse immunoglobulin (Ig) G, and is vulnerable to interference from human antibodies with reactivity to animal antibodies, such as human anti-mouse antibodies or heterophilic antibodies [1–3]. Rheumatoid factor (RF) is a group of autoantibodies with reactivity to the Fc of human IgG, and may behave as heterophilic antibodies by cross-reacting with antibodies from other species [4, 5]. RF and heterophilic antibodies have the potential to cause falsely elevated test results by cross-linking the assay antibodies, even in the absence of analyte, most often via binding to the Fc-part of assay antibodies [6].

A much publicised case from the late 1990s illustrates the potential consequences when incorrect test results caused by interference lead to mismanagement of patients [7]. Repeated elevated results for human chorionic gonadotropin (hCG) in a young, non-pregnant woman, misled her gynaecologists to suspect trophoblastic disease. In addition to being used as a pregnancy marker, hCG is an important tumour marker, primarily in testicular cancer and trophoblastic disease [8, 9]. The young woman was subjected to several chemotherapy regimens, then hysterectomy, bilateral salpingo-oophorectomy and thoracotomy. No malignant disease was found in biopsies or surgical resectates, her hCG remained unchanged and was shown to be falsely elevated when a sample was sent for analysis in a different hCG-assay. In addition to harming patients, false test results also have the potential to confound research results.

Most modern immunoassays are designed with specific protective measures against interference from RF and heterophilic antibodies. However, despite available knowledge and tools to limit interference, not all commercial immunoassays have sufficient protection. A study performed by Bolstad et al in 2011, showed that 21 out of 170 commercial immunoassay kits tested were susceptible to interference from heterophilic antibodies [3]. In addition, these patient antibodies are diverse entities that may be present in high concentrations, and interference may occur despite protective measures [6].

Previous studies have revealed that RF is associated with interference in multiplex cytokine assays mostly used for research purposes [5, 10–12], but less is known regarding immunoassays used in clinical practice. Based on published data, but also on our own experience from immunoassay and interference research, we believe that interference from RF is a larger problem than what is commonly acknowledged among clinicians treating patients with RA. To our knowledge, interference from RF in immunoassays commonly used in clinical practice and research has not been studied in a large cohort of early arthritis patients. Furthermore, it is difficult to predict which patient samples are most susceptible to interference. High levels of RF are often considered a risk factor, but there are limited data to support this association [10, 12].

The main aims of this study were to assess the prevalence of RF reactivity to animal antibodies and to test if selected commercial immunoassays are vulnerable to interference from RF-positive sera from an early arthritis population. We also wanted to identify predictors for immunoassay interference in RA-patients.

## Methods

### The Norwegian Very Early Arthritis Clinic (NOR-VEAC) and sample selection

The NOR-VEAC study is a prospective observational cohort including patients from six rheumatology departments in Norway [13]. Patients aged 18–75 years with ≥ 1 swollen joint(s) of ≤ 16 weeks’ duration were eligible for inclusion. Clinical data and samples were collected at baseline, 3, 6, 12 and 24 months. For the current analyses, we included samples and clinical data from patients with a final clinical diagnosis of RF-positive RA enrolled in the study from 2004 to 2010. Patients with RF-negative RA or psoriatic arthritis (PsA) from the same cohort, were included as controls. The serum samples were stored at −70 °C. We aimed to include one sample collected prior to the initiation of disease modifying antirheumatic drug (DMARD) for all patients. To assess the effect of treatment on RF reactivity, we also selected one sample collected approximately 3 months after the initiation of DMARD for the RF-positive RA patients with available sample both prior to and after initiation of DMARD.

### Clinical outcome measures

The 28-joint disease activity score-erythrocyte sedimentation rate (DAS28-ESR) was the selected disease activity measure [14].

### Measurement of RF

Results from previous analyses of RF IgM, RF IgA and anticyclic citrullinated peptide were used in the current study [15]. The assay has been described elsewhere and is used both in clinical practice and previous publications [15–17]. Patients were classified as RF-positive if IgM and/or IgA RF ≥ 25 IU/mL. Details are provided in Supplementary Appendix S1.

### Characterisation of RF cross-reactivity to animal antibodies

Reactivity to mouse IgG1, mouse IgG2a, rabbit IgG, bovine IgG, sheep/goat IgG and human IgG was analysed using in-house interference assays. Interference assays are non-sense assays, using intact IgGs with no common antigen, as capture and tracer antibodies [18]. A positive signal indicates cross-linking of the assay antibodies by interfering patient antibodies. We used three-step immunofluorometric assays automated on the AutoDELFIA (PerkinElmer, Waltham, MA, USA) immunoassay platform [19]. The assays (except the bovine IgG assay) were run with blocking bovine immunoglobulin in the assay buffer to reduce the risk of false positive results caused by cross-reactive anti-bovine Ig, which are common in the normal population [20]. The results were reported in arbitrary units per litre (AU/L) and results exceeding 200 AU/L were truncated. Values > 10× blank serum were defined as positive with regard to reactivity. The cut-off was set using a pragmatic approach*,* based on our experience using interference assays to identify samples with risk of interference in routine immunoassays. High levels were defined as values above the 75th percentile of the RF-positive RA patients.

### Testing of interference in commercial immunoassays

Samples showing strong reactivity against mouse IgG1 were selected for testing in three commercial immunoassays previously shown to be susceptible to interference from endogenous antibodies; the Abbott Architect Total β-hCG assay (Abbott Diagnostics, Abbott Park, IL), the BioRad 27-plex cytokine assays (Bio-Rad Laboratories, Hercules, CA) and the Roche Elecsys Soluble Transferrin Receptor (sTfR) assay (Roche Diagnostics, Mannheim, Germany) [3, 10, 12]. To reveal interference, values obtained before and after addition of blocker (aggregated murine IgG1 PolyMAK (Roche)) were compared. For β-hCG we also compared values from two different assays; the Abbott Architect β-hCG assay and the Roche Elecsys hCG + β assay. For all three tested assays (β-hCG-, 27-plex cytokine- and sTfR assays), interference was defined as a discrepancy between the unblocked and blocked value, likely to influence clinical interpretation of the results, and exceeding the reported assay imprecision with a considerable margin. Details are provided in the Supplementary Appendix S1.

### Statistical analyses

Between-group comparisons were assessed using independent samples *t* test, Mann–Whitney *U* test or χ^2^ test, as appropriate. Statistical tests were two-sided with level of significance set at *p* < 0.05.

Correlations between RF IgA and/or IgM and reactivity against animal and human antibodies, as well as pretreatment disease activity, were examined using linear regression analyses and Pearsons correlations. Further, the association between disease activity and anti-animal and anti-human IgG reactivity was assessed using multivariable logistic regression analyses, adjusting for RF IgM and RF IgA. Statistical analyses were performed using IBM SPSS Statistics, Version 25.

## Results

### Study population and sample selection

**Fig. 1:**
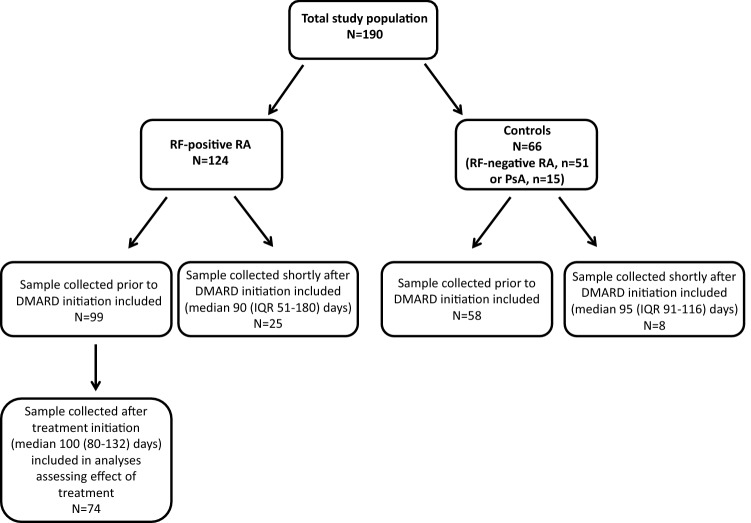
Overview of study population and included samples. *DMARD* disease-modifying antirheumatic drug; IQR interquartile range; *PsA* psoriatic arthritis; *RA* rheumatoid arthritis; *RF* rheumatoid factor

Samples and clinical data from 124 patients with RF-positive early RA and 66 controls (RF-negative RA (*n* = 51) or PSA (*n* = 15)), were included in the study. An overview of the study population and included serum samples is provided in Fig. 1. Among the RF-positive RA patients, DMARD treatment was initiated in 113 out of 124 patients. Baseline demographics and clinical characteristics were similar in the RF-positive RA group and the control group (Table 1).

**Table 1:** Baseline characteristics in RA-positive RA and control group

	RF-positive RA (*n* = 124)	Controls (*n* = 66; 51 RF-neg RA, 15 PsA)
Age, years, mean (SD)	50 (13)	51 (13)
Female, *n* (%)	75 (61)	45 (67)
BMI, mean (SD)	26 (4)	25 (5)
DAS28, mean (SD)	5.0 (1.3)	5.0 (1.5)
PGA, mean (SD)	56 (24)	57 (23)
SJC28, mean (SD)	5.8 (4.5)	8.3 (6.4)
TJC28, mean (SD)	6.7 (5.3)	7.8 (7.4)
SJC68, mean (SD)	9.2 (7.0)	10.4 (8.6)
ESR (mm/h), median (IQR)	28 (14–48)	28 (12–48)
CRP (mg/L), median (IQR)	14 (5–34)	19 (7–44)
RF IgA (IU/mL), median (IQR)	36 (17–77)	3 (1–6)
RF IgM (IU/mL), median (IQR)	90 (44–180)	3 (1–6)
ACPA-positive, *n* (%)	105 (85)	18 (27)
ACPA (IU/mL), median (IQR)	204 (54–315)	3 (2–34)

*RF* rheumatoid factor; *RA* rheumatoid arthritis; *PsA* psoriatic arthritis; *BMI* body mass index; *DAS28* 28-joint Disease Activity Score; *PGA* Patient Global Assessment; *SJC28* 28-joint swollen joint count; *TJC28* 28-joint tender joint count; *ESR* erythrocyte sedimentation rate; *CRP* C-reactive protein; *ACPA* anticyclic citrullinated peptide; *SD* standard deviation; *IQR* interquartile range

### RF reactivity to animal antibodies

**Fig. 2:**
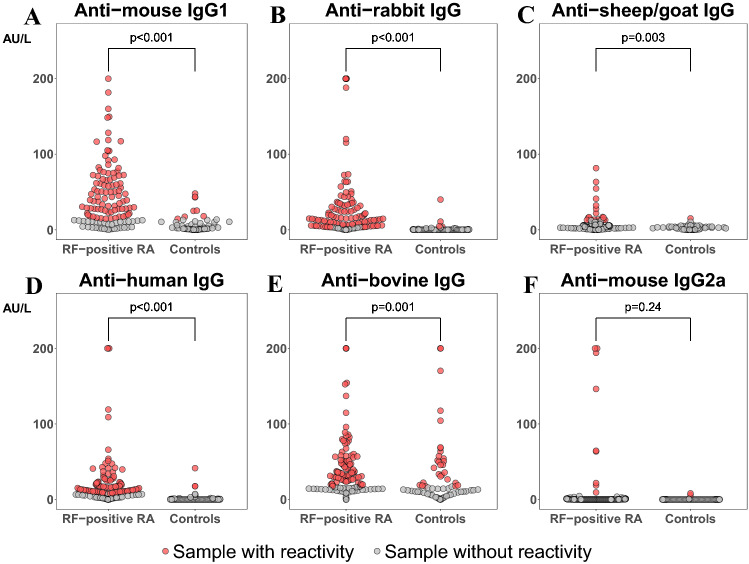
**a**–**f** Sample reactivity against animal and human antibodies. Rheumatoid factor (RF)-positive rheumatoid arthritis (RA) patients (*n* = 124), compared to controls (RF-negative RA and psoriatic arthritis patients) (*n* = 66). **a** Anti-mouse IgG1 reactivity. **b** Anti-rabbit IgG reactivity. **c** Anti-sheep/goat IgG reactivity. **d** Anti-human IgG reactivity. **e** Anti-bovine IgG reactivity. **f** Anti-mouse IgG2a reactivity. *p* value < 0.05 indicates a statistically significant difference in proportion of samples with reactivity in the RF-positive RA group vs. the control group

Results from all six interference assays were obtained in 98% (*n* = 188) of patients. As shown in Fig. 2a–f, we found considerably more reactivity toward animal antibodies in sera from RF-positive RA-patients, compared to the control group. Notably, anti-mouse IgG1 reactivity was found in 73% of patients with RF-positive RA and 12% of controls (*p* < 0.001).

### Results from interference testing in commercial immunoassays

#### β-hCG assay

**Fig. 3:**
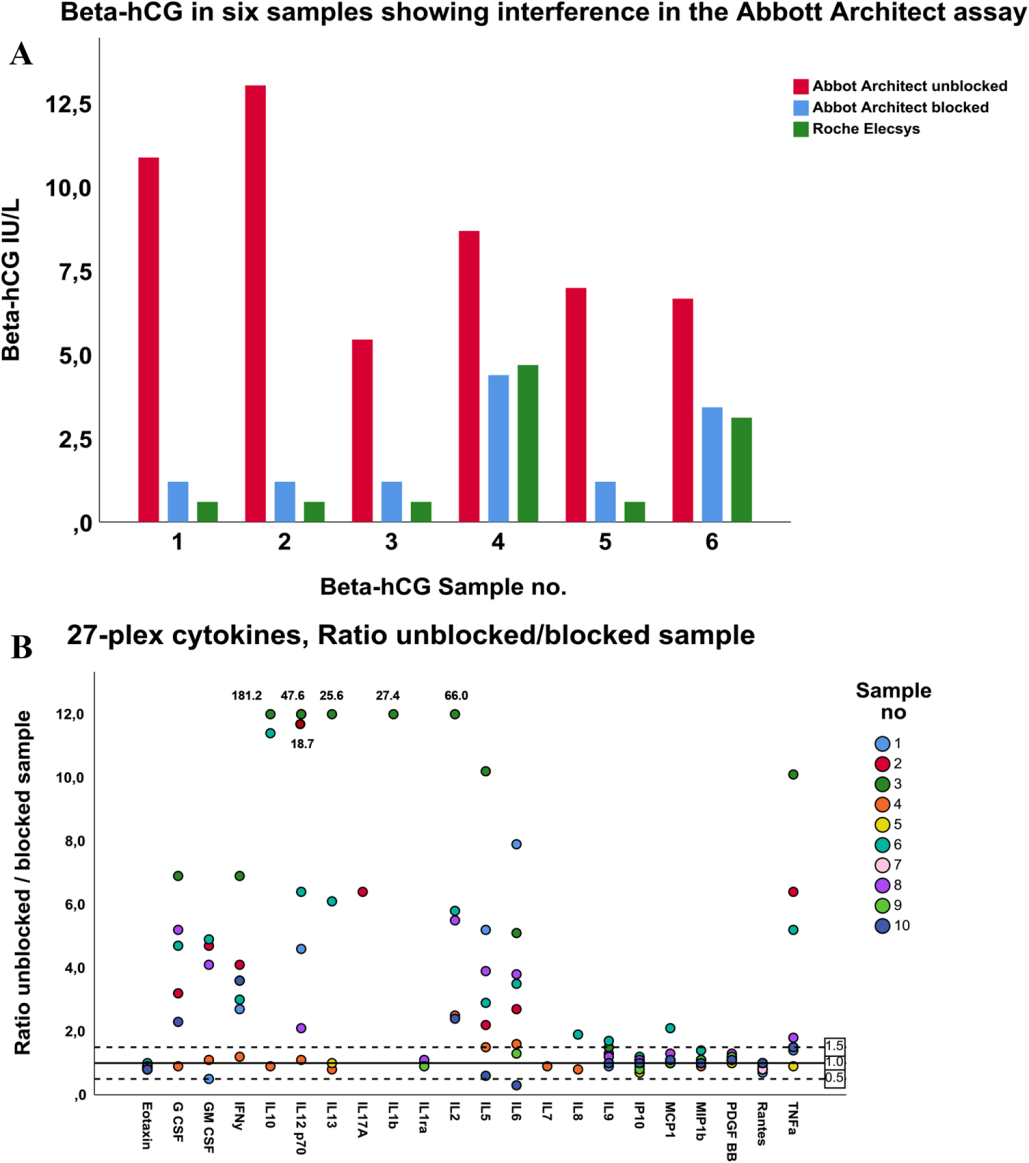
**a**, **b** Test of samples for interference in commercial immunoassays. **a** Beta-hCG in six samples showing interference in the Abbott Architect Total β-hCG assay. The lower reporting limit was 1.2 IU/L in the Architect Total β-hCG assay and 0.1 IU/L in the Elecsys hCG + β assay. **b** Ratio between results for the paired unblocked and blocked samples from ten patients in the BioRad 27-plex cytokine assay. Interference was defined as a ratio between unblocked/blocked samples > 1.5 or < 0.5 in combination with result of unblocked sample above the fourth lowest calibrator. Ratios > 12 were truncated. IL4, Basic FGF, IL15, MIP1a and VEGF had no values above the fourth lowest calibrator, and are not shown

Six out of 31 sera yielded considerably higher values in the Abbott Architect β-hCG assay than in the Roche Elecsys hCG + β assay, and were consequently selected for blocking. Interference was shown in all six samples (Fig. 3a), which corresponds to 19% (6/31) of the tested samples or 5% (6/124) of the RF-positive RA population overall. β-hCG in unblocked sera ranged from 5.4 to 13.0 IU/L in the Architect β-hCG assay, and were 3.2–12.0 IU/L higher than in blocked samples. The values obtained in the Elecsys hCG + β assay were similar to the values obtained in the Architect β-hCG assay after blocking. The median (IQR) level of RF IgA was 46 (28–66) IU/mL and RF IgM was 199 (111–266) IU/mL, in the six patients with interference.

#### 27-plex cytokine assays

Interference was demonstrated in seven out of 10 sera (for 2–13 cytokines), analysed in the BioRad 27-plex cytokine assays (Fig. 3b). Large discrepancies in test results between the unblocked and paired blocked samples were revealed. Moreover, 15 out of the 27 cytokine assays were found to be susceptible to interference.

#### Soluble serum Transferrin Receptor (sTfR) assay

Our results showed interference in two out of 33 samples tested in the Roche Elecsys sTfR assay. In these, the unblocked samples yielded sTfR values of 8.1 and 8.2 mg/L, vs. 4.2 and 6.0 mg/L in blocked samples, respectively. Patient 1 had RF IgA of 26 IU/mL and RF IgM 132 IU/mL, and patient 2 had RF IgA of 26 IU/mL and RF IgM 67 IU/mL.

### Possible predictors of reactivity and interference in patients with RF-positive RA

#### Level of RF IgA and/or IgM

We examined whether RF IgA and IgM were correlated with anti-animal and human IgG reactivity. Weak associations between RF IgA and anti-rabbit IgG, as well as between RF IgM and anti-mouse IgG1, anti-rabbit IgG and anti-human IgG, were found (Table 2).

**Table 2:** Correlations between rheumatoid factor IgA and IgM and reactivity against animal and human antibodies

	RF IgA			RF IgM		
	*R*	β (95% CI)	*p*	*R*	β (95% CI)	*p*
Anti-mouse IgG1	0.13	0.06 (−0.02–0.14)	0.14	0.37	0.13 (0.07–0.19)	< 0.001
Anti-rabbit IgG	0.23	0.12 (0.03–0.22)	0.01	0.54	0.23 (0.17–0.30)	< 0.001
Anti-human IgG	0.07	0.03 (−0.04–0.09)	0.47	0.28	0.08 (0.03–0.13)	0.002

Linear regression analyses (using Pearson’s correlation). *RF* rheumatoid factor; *R* correlation coefficient, *β* slope coefficient; *CI* confidence interval

#### Disease activity

We also examined whether disease activity prior to initiation of DMARD was associated with reactivity against animal and human IgG. As shown in Table 3, more patients with DAS28 > 3.2 showed reactivity against mouse IgG1 and human IgG, compared to patients with DAS28 ≤ 3.2. Pretreatment DAS28 was not correlated to RF IgA or RF IgM levels, β −5.0 (95% CI −16.6, 6.6), *p* = 0.39, and β −6.7 (95% CI −20.9, 7.5), *p* = 0.35, respectively.

**Table 3:** Proportion of patients with reactivity against animal and human IgG, stratified by DAS28 > 3.2 vs. ≤ 3.2 prior to DMARD initiation

Antibody reactivity, *n* (%)			
	DAS28 > 3.2 *n* = 90	DAS28 ≤ 3.2 *n* = 27	OR (95% CI)*, *p*
Anti-mouse IgG1	69 (77%)	14 (52%)	4.4 (1.6–12.2), *p* = 0.005
Anti-rabbit IgG	76 (84%)	19 (70%)	3.3 (1.1–10.1), *p* = 0.04
Anti-human IgG	67 (75%)	11 (41%)	8.5 (2.7–27.1), *p* < 0.001
Anti-bovine IgG	62 (69%)	16 (59%)	1.6 (0.7–3.9), *p* = 0.30

*DAS28* Disease Activity Score 28 joints; *OR* odds ratio; *CI* confidence interval

^*^Odds ratio (95% confidence interval) (logistic regression analyses adjusted for RF IgM and RF IgA) for antibody reactivity in RF-positive RA patients with DAS28 > 3.2 vs. ≤ 3.2. DAS28 score was available in 117 out of 124 patients

### Effect of DMARD-treatment on levels of anti-mouse IgG1 and anti-rabbit IgG

Furthermore, we examined whether the levels of anti-mouse IgG1 and anti-rabbit IgG reactivity declined after start of treatment, among the 74 RF-positive RA patients who had paired samples from before and after DMARD initiation. In patients with high reactivity (≥ 65 AU/L) against mouse IgG1 prior to treatment, the levels declined after treatment initiation in 19 out of 22 patients (mean difference −35 (95% CI −44 to −26) AU/L). Eleven out of 14 patients with high levels of anti-rabbit IgG (≥ 33 AU/L) had lower levels after treatment initiation (mean difference −64 (95% CI −110 to −18) AU/L).

## Discussion

We have assessed the prevalence of anti-animal and anti-human IgG-reactivity and the associated risk of interference in immunoassays commonly used in clinical care and research, in a large cohort of recent-onset RA patients. Our study revealed a high prevalence of reactivity against animal and human IgG in sera from RF-positive RA patients. Moreover, interference was demonstrated in a considerable proportion of samples in the Abbott hCG and the BioRad 27-plex cytokine assays. With regard to possible predictors of vulnerability to interference, we found weak associations between both level of RF IgA and IgM, as well as disease activity, and anti-animal IgG reactivity in our cohort. Further, we found that the degree of anti-IgG reactivity was moderately reduced after treatment initiation in patients with high reactivity prior to treatment. Our study showed that immunoassay interference continues to be a problem in RF-positive RA patients, despite available knowledge and tools to prevent it.

The remarkably high prevalence of anti-animal IgG reactivity revealed in this cohort, demonstrated that RF reactivity is not restricted to human IgG only. The ability of RF to cross-react with IgG from other species has also been shown in previous studies, but these included few RF-positive RA patients [4, 5]. The high degree of reactivity against mouse and rabbit IgG is important, because these are the most common animal antibodies in immunoassays. Sheep antibodies are used less frequently overall, but are preferred by some immunoassay developers. As expected, anti-bovine IgG reactivity was quite common in both the RF- and the control group. It is well-known that anti-bovine reactivity is common in the normal population [20], and most assays are protected against interference from anti-bovine reactivity.

Interference was revealed in 19% of the tested sera (5% of total) in the Abbott Architect β-hCG assay. Considering that the risk of interference from heterophilic antibodies in this assay has been known for decades [3, 7], and the considerable resources available to the company, the high rate of interference is surprising. As mentioned in the Introduction, β-hCG is an important tumour marker, in addition to a marker of pregnancy [8, 9]. According to the package insert of the Architect β-hCG assay, the assay is only intended for use in the early detection of pregnancy [21]. However, we believe that the Architect β-hCG assay is still used in management of patients with suspected or established malignant disease, because many clinicians ordering the test do not know which assay is used or are not aware of the limitations of this assay.

In the 27-plex cytokine assay, our study revealed interference in 70% of tested sera, and 17 out of 27 cytokine assays. In line with other publications, our results showed that interference from RF can be reduced by addition of blocking agents [10–12]. We encourage the assay producer to protect these assays against interference from RF, e.g. by adding appropriate blocking agents to assay reagents, as cytokines are highly relevant research biomarkers in this patient group.

The sTfR assay is commonly used in diagnosis of iron deficiency anaemia in RA-patients, due to s-ferritin being unspecific in the context of inflammation [22, 23]. In spite of this, interference from RF was still a problem in this assay [3]. RF levels were only moderately elevated in the two patients with interference in this assay.

We aimed to identify possible predictors of assay interference in RA patients. We found that being RF-positive is a clear predictor of assay interference, which is known among immunoassay developers and often listed as a limitation in assay package inserts. However, levels of RF IgA and IgM were only weakly correlated with the level of anti-animal IgG reactivity in our study, suggesting that antibody properties such as avidity and specificity could be equally important. In line with our results, other studies have shown that interference in multiplex cytokine assays is unpredictable in relation to RF level, and all sera from RA patients should be treated as likely to interfere in multiplex assays [10, 12].

Although our results showed a decline in anti-IgG reactivity after treatment initiation among patients with high reactivity prior to treatment, there was still considerable reactivity in the samples collected after start of treatment. Consequently, we believe that although the risk of interference is probably highest before starting treatment, the risk of interference does not disappear after initiation of effective therapy.

It is important that clinicians are alert to the risk of immunoassay interference in RA patients. Clinicians should contact their laboratory in cases of unexpected laboratory results. Most laboratories have available strategies to investigate samples with suspected interference. These strategies include reanalysis with an alternative method, sample dilutions, addition of blocking reagents and antibody depletion (PEG, ammonium sulphate etc.) [6]. Most importantly, effective measures to protect the assays should be taken by commercial producers during assay development [2, 24]. Adding on to the risk of interference from RF, RA patients and other patients with chronic diseases have an increased risk of false test results due to accumulation of many laboratory tests over time. This further illustrates the importance of only ordering clinically necessary tests.

The main strength of our study was the use of clinical data from a relatively large and well-characterised real-life cohort of patients, both before and after initiation of DMARD treatment, leading to applicability of our results both in regular clinical care and research. The main limitation of our study was that we did not test all samples in the commercial immunoassays, to know the true prevalence of interference in our cohort, but we had to prioritise because of practical and economic limitations, especially with regard to the expensive 27-plex cytokine assay. Testing in additional assays could probably provide valuable information regarding the occurrence of RF interference in commercial immunoassays. Previous studies have revealed clinically relevant discrepancies in results obtained by different RF assays, which could theoretically influence the stratification of patients with regard to RF status [25, 26].

In conclusion, our study revealed considerable reactivity to animal antibodies in RF-positive RA patients, with a worrying rate of falsely elevated test results in immunoassays used in clinical care and research. Reactivity was only weakly associated with RF level and disease activity, and moderately reduced by treatment initiation. False test results may confound research, but also lead to potentially harmful diagnostic and therapeutic interventions in patients. Physicians as well as researchers, laboratories and assay manufacturers must be alert to the risk of falsely elevated test results in RF-positive RA patients. This is particularly important when results are unexpected or discordant with clinical findings.

## Supplementary Information

Below is the link to the electronic supplementary material.

Supplementary file1 (DOCX 23 KB)

## Data Availability

The dataset used and analysed during the current study is available from the corresponding author on reasonable request.

## Abbreviations

AU/L: Arbitrary units per litre
DAS28-ESR: 28-Joint disease activity score-erythrocyte sedimentation rate
DMARD: Disease modifying antirheumatic drug
hCG: Human chorionic gonadotropin
Ig: Immunoglobulin
NOR-VEAC: Norwegian Very Early Arthritis Clinic
PsA: Psoriatic arthritis
RA: Rheumatoid arthritis
RF: Rheumatoid factor
sTfR: Soluble transferrin receptor
