# Supplementary material for: Rheumatoid factor and falsely elevated results in commercial immunoassays: data from an early arthritis cohort
Source: Rheumatol Int. 2021 May 4;41(9):1657–65. doi: 10.1007/s00296-021-04865-9 (PMC8316178; doi:10.1007/s00296-021-04865-9)
Supplement: Supplementary file 1 — Supplementary file1 (DOCX 23 KB) [file 296_2021_4865_MOESM1_ESM.docx]

# Supplementary appendix S1

**Methods**

**Measurement of rheumatoid factor and anticyclic citrullinated peptide**

Rheumatoid factor (RF) IgM and IgA were measured in stored sera with an in-house ELISA using purified rabbit IgG Fc as solid phase and enzyme-conjugated goat anti-human IgA and IgM F(ab’)2 as tracer, automated on the Freedom EVOlyzer 2 (1). Anticyclic citrullinated peptide was assessed by the anticyclic citrullinated peptide 2 test (Inova Diagnostics, San Diego, California, USA, and Phadia, Freiburg, Germany).

**Testing of interference in commercial immunoassays; description of blocking and definitions of interference in the three assays**

Abbott Architect Total β-hCG assay:

Samples assayed in the Architect Total β-hCG assay were selected for reanalysis after addition of blocker, if the unblocked value was >5 IU/L (upper reference limit) and/or the value in the Architect Total β-hCG was >50% higher than in the Roche Elecsys hCG+ β assay. The Elecsys hCG+ β assay has previously been shown to be more resistant to interference from Fc-reactive heterophilic antibodies (2). Interference in the Architect Total β-hCG assay was defined as: Value of >5.0 IU/L in unblocked sample and a difference >50% between the unblocked and blocked sample.

BioRad 27-plex cytokine assays:

All 10 samples were assayed in pairs, as unblocked and blocked samples. Interference was defined as a ratio between unblocked/blocked samples >1.5 or < 0.5 in combination with result of unblocked sample above the fourth lowest calibrator.

Roche Elecsys Soluble Transferrin Receptor (sTfR) assay:

All samples were tested both before and after addition of blocker. Interference was defined as: Value >4.4 mg/L in women and >5.0 mg/L in men (upper limit of reference interval) in unblocked samples and a difference of >25% between the unblocked and blocked sample.

**Results**

RF IgA and IgM had been measured in a sample collected at baseline in 94% of patients, and at the 3-month visit in the remaining 6%.

**References**

1. Mjaavatten MD, van der Heijde DM, Uhlig T, Haugen AJ, Nygaard H, Bjørneboe O, et al. Should anti-citrullinated protein antibody and rheumatoid factor status be reassessed during the first year of followup in recent-onset arthritis? A longitudinal study. The Journal of rheumatology. 2011;38(11):2336-41.

2. Bolstad N, Warren DJ, Bjerner J, Kravdal G, Schwettmann L, Olsen KH, et al. Heterophilic antibody interference in commercial immunoassays; a screening study using paired native and pre-blocked sera. Clinical chemistry and laboratory medicine. 2011;49(12):2001-6.
